# Overspecialized and undertrained? Patient diversity encountered by medical students during their internal medicine clerkship at a university hospital

**DOI:** 10.1186/s12909-015-0353-y

**Published:** 2015-03-31

**Authors:** Simon Melderis, Jan-Philipp Gutowski, Sigrid Harendza

**Affiliations:** III. Medizinische Klinik, Universitätsklinikum Hamburg-Eppendorf, Martinistraße 52, 20246 Hamburg, Germany

**Keywords:** Internal medicine, Clerkship, Patient diversity, Patient encounter cards, Medical education

## Abstract

**Background:**

During the four-month internal medicine clerkship in their final year, undergraduate medical students are closely involved in patient care. Little is known about what constitutes their typical learning experiences with respect to patient diversity within the different subspecialties of internal medicine and during on call hours.

**Methods:**

25 final year medical students (16 female, 9 male) on their internal medicine clerkship participated in this observational single-center study. To detail the patient diversity encountered by medical students at a university hospital during their 16-week internal medicine clerkship, all participants self-reported their patient contacts in the different subspecialties and during on call hours on patient encounter cards. Patients’ chief complaint, suspected main diagnosis, planned diagnostic investigations, and therapy in seven different internal medicine subspecialties and the on call medicine service were documented.

**Results:**

496 PECs were analysed in total. The greatest diversity of chief complaints (CC) and suspected main diagnoses (SMD) was observed in patients encountered on call, with the combined frequencies of the three most common CCs or SMDs accounting for only 23% and 25%, respectively. Combined, the three most commonly encountered CC/SMD accounted for high percentages (82%/63%), i.e. less diversity, in oncology and low percentages (37%/32%), i.e. high diversity, in nephrology. The percentage of all diagnostic investigations and therapies that were classified as “basic” differed between the subspecialties from 82%/94% (on call) to 37%/50% (pulmonology/oncology). The only subspecialty with no significant difference compared with on call was nephrology for diagnostic investigations. With respect to therapy, nephrology and infectious diseases showed no significant differences compared with on call.

**Conclusions:**

Internal medicine clerkships at a university hospital provide students with a very limited patient diversity in most internal medicine subspecialties. Shadowing the on call resident or shorter rotations could provide a more extended patient diversity.

## Background

General internal medical diseases such as hypertension, diabetes or renal insufficiency are highly prevalent in adults [1] and will be even more relevant in the future due to an aging population in many countries [2,3]. They can interfere with the management of other, unrelated problems, e.g. perioperative care [4,5] or anaesthesia [6] and require an acquaintance with multiple medications from all medical disciplines [7]. Therefore, all medical school graduates need to be equipped with the necessary knowledge and skills to deal with common internal medical conditions, independently of their discipline of choice for postgraduate training.

On the other hand, medical knowledge and treatment options are growing ever more complex [8] and subspecialization is inevitable, especially in internal medicine [9]. As early as the 1980s, specialty training and board certification in a certain internal medicine subspecialty became the norm in America [10]. In Germany, particularly at university and other teaching hospitals, internal medicine departments are subspecialized. Hence, depending on the specific curriculum at a given medical school, the majority of the undergraduate teaching is delivered by specialists, who will often only cover their area of expertise. If learning objectives for general internal medicine are not well defined, these circumstances could lead to a lack of general internal medicine training.

Junior doctors from Ireland reported in a survey that they felt inadequately prepared for internship by medical school [11]. A study amongst German physicians in their first and second year of postgraduate training revealed perceptions of unpreparedness with respect to general internal medicine skills such as EKG interpretation as well as treatment and therapy planning [12]. Those skills are especially needed for patient encounters during on call hours when diagnoses have to be made quickly to initiate emergency treatment [13].

In the final year of their six-year undergraduate training medical students in Germany are required to complete a four-month hospital based rotation in internal medicine [14]. During that time on the wards, they clerk patients and learn the day-to-day practical tasks of being a physician. We hypothesize that the specific patients they encounter in the different subspecialties of internal medicine greatly influence their learning experiences. To our knowledge there has been no study detailing the nature of these different internal medicine patient encounters in undergraduate medical education. Furthermore, it is not known whether learning experiences in internal medicine with respect to patient encounters differ between learning on the wards and whilst on call. It has been demonstrated though that great differences in clerkship experiences exist between family medicine, paediatrics and internal medicine [15]. In postgraduate medical education, subspecialty elective exposures were without impact on outcomes on the American board of internal medicine certification examination [16].

In order to analyze how well graduates are being prepared in dealing with common internal medical problems, we characterized the patient diversity encountered by final year students during their internal medicine clerkship. Our research questions were: are clerkships in different subspecialties of internal medicine at a university hospital associated with a more limited education in general internal medicine? Do patient encounters during on call periods differ from those on the specialized wards? Our primary objective was to quantify, qualify and compare the spectrum of conditions within the various subspecialties of internal medicine including those during on-call hours that students encounter during their final year clerkship.

## Methods

### Setting

At Hamburg University Medical Center the four month final year internal medicine clerkship is split into two rotations of eight weeks. For these, depending on availability, each student can choose two of the following subspecialties of internal medicine at our university hospital: cardiology, endocrinology, gastroenterology, infectious diseases, nephrology, oncology/hematology, and pulmonology. The medical content of the internal medicine rotation is currently not regulated or standardized. The students perform supervised work, similar to that of interns on internal medicine wards. They are specifically encouraged to clerk in new patients. Shadowing the on call internal medicine resident had not been part of the regular clerkships so far.

### Participants

All final year medical students who started their internal medicine clerkship in the July and December 2013 cohorts at Hamburg University Medical Center were invited to participate in this study. Participation was voluntary and prior to enrolment participating students gave informed consent. They could leave the study at any time without giving any reasons.

### Patient encounter cards (PECs)

Based on patient encounter cards, which included age and sex of the patient as well as location of the encounter, level of involvement or supervision, diagnostic procedures, principal diagnosis (including aetiology, organ system involvement, and severity of complications), and up to four secondary diagnoses [15], and post-encounter forms [17], PECs were developed for students in their final year internal medicine rotation to document the patients they encountered during their work on the wards. Each student created a unique code for pseudonymization. Parameters for documentation included: 1) patient demographic information such as age and sex, 2) chief complaint (CC), 3) suspected main diagnosis (SMD), 4) up to four relevant secondary diagnoses, 5) planned investigations (PI), and 6) planned therapy (PT). Students were given written and oral information on how to fill in the PECs and any remaining questions were addressed during the first week of the study. Initially participants were provided with 20 PECs, to be carried in the pockets of their white coats. They were asked to document every patient encounter they considered significant and in which they were involved with respect to clerking and/or participating in management decisions regarding diagnostic and/or therapeutic considerations. Students were asked to complete the PECs to the best of their knowledge without help from staff or supervisors. The PECs were collected two to three times per week, and students were provided with an additional supply of PECs when needed.

### On call

Students were encouraged to shadow the internal medicine on call resident. These shifts lasted from 5 pm until 10 pm on weekdays and students were given compensatory time off on the wards. On call, PECs were identical to normal PECs but yellow in color. At Hamburg University Medical Center the out-of-hours internal medicine on call resident is responsible for all patients admitted under internal medicine as well as patients admitted under other specialties who present with additional internal medical problems. Typical activities range from dealing with emergencies (e.g. pulmonary edema) to checking results of blood tests.

### Ethics

The study was performed in accordance with the Declaration of Helsinki and a member of the Ethics Committee of the Chamber of Physicians, Hamburg, confirmed the innocuousness of the study protocol.

### Analyses

**Figure 1:**
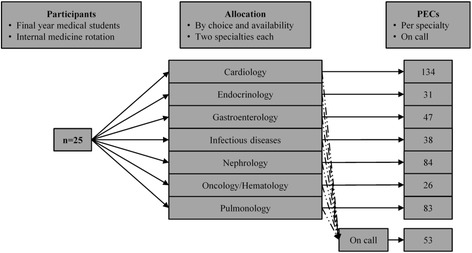
**Study design: allocation of participants to the subspecialties and the on call “rotation”.** Total number of PECs collected per subspecialty and during on call.

We used an observational study design where the unit of observation was a single student-patient interaction, as documented by the PECs, and the unit of analysis was the combined data of all these interactions/PECs within the different subspecialties (Figure 1). For comparison of the different subspecialties, we grouped all PECs according to the subspecialties (cardiology, endocrinology, gastroenterology, infectious diseases, nephrology, oncology/hematology, and pulmonology) to which the students were assigned. All PECs that were completed during on calls were grouped together and handled as a separate “subspecialty”.

Prior to data collection, chief complaints (CCs) were categorized based on our faculty’s clerkship logbook which includes a list of common chief complaints [18]. Suspected main diagnoses (SMDs) were classified based on the ICD-10 system [19]. We grouped CCs and SMDs according to clinical judgement of the authors. For example, all primary lung cancers were put into a combined group, irrespective of histology or exact site. SM and PG grouped CCs independently and SH was involved in the discussion when no consensus on the grouping of a CC could be reached.

We also developed a blueprint of commonly encountered diagnostic procedures prior to data collection. The diagnostic procedures ranged from simple bed side tests (e.g. pulse oximetry) to invasive diagnostic procedures (e.g. intracardiac catheter). After completion of the study, ambiguous answers were discussed (PG, SM) and manually assigned to a category. Upon data analysis, any diagnostic procedure or therapy that was not pre-categorized was either added to a fitting category or grouped as a new category according to clinical judgement. Each diagnostic procedure was categorized as either basic (e.g. pulse oximetry) or non-basic (e.g. cardiac catheterization) according to our faculty’s clerkship logbook [18] involving a similar process of discussion as mentioned above when no consensus could be reached about how to categorize a diagnostic procedure. Planned therapies were categorized analogous to diagnostic procedures.

### Statistical methods

Data for study participants and PECs collected are expressed as mean ± standard deviation. Student’s *t*-test was used to compare means. Chief complaints, suspected main diagnosis, investigations and therapies are expressed as percentage of all completed PECs collected within a subspecialty. The chi-squared test was used to explore the relationship between frequencies and subspecialties. Statistical analysis was performed using Excel (Microsoft®) and GraphPad Prism version 6.04 for Windows, GraphPad Software, San Diego California USA. P < 0.05 was considered statistically significant.

## Results

### Demographics

All 25 students (16 female, nine male) from the two cohorts who were approached participated in the study and completed it. The mean age of all participants was 26.4 ± 2.1 years (female: 26.4 ± 2.2; male: 26.3 ± 2.1). We collected and analysed a total of 496 PECs. Each student completed on average 19.8 PECs ± 10.0 (female: 19.6 ± 11.0; male: 22.2 ± 8.5). There was no statistically significant difference between the sexes.

### Chief complaints

**Figure 2:**
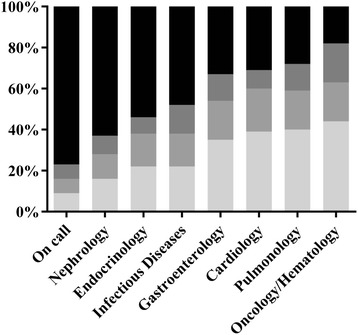
**Diversity of chief complaints (CC): chief complaints (% of total complaints); columns from top to bottom: 1st most frequent CC (light grey), 2nd most frequent CC (medium grey), 3rd most frequent chief complaint (dark grey), other CCs (black).**

We found differences in both the diversity and nature of the chief complaints encountered within the different subspecialties. The greatest diversity was observed in the on call patients where the three most common chief complaints together accounted for only 23% of all on call PECs (Figure 2). On the wards, the three most common CCs ranged from 37 to 82%. The three most commonly encountered CCs were for the subspecialties: nephrology: fatigue and B symptoms, dyspnea, urogenital; endocrinology: admitted-with-diagnosis, fatigue and B symptoms, dyspnea/ musculoskeletal; infectious diseases: admitted-with-diagnosis, fatigue and B symptoms, fever; gastroenterology: admitted-with-diagnosis, abdominal pain, abnormal liver function tests; oncology/hematology: fatigue and B symptoms, admitted-with-diagnosis, musculoskeletal; pulmonology: dyspnea, cough, fatigue and B symptoms; cardiology: dyspnea, chest pain, admitted-with-diagnosis. The most commonly encountered complaint on call was abdominal pain.

### Suspected main diagnosis

**Figure 3:**
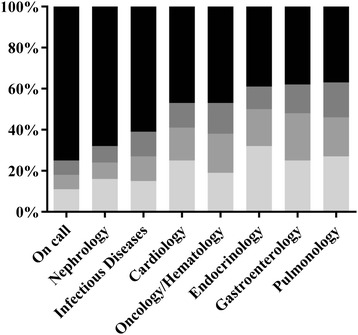
**Diversity of suspected main diagnoses (SMDs): columns from top to bottom: 1st most frequent SMD (light grey), 2nd most frequent SMD (medium grey), 3rd most frequent SMD (dark grey), other SMDs (black).**

The percentage of the three most commonly encountered suspected main diagnoses (SMDs) per subspecialty or on call, respectively, is shown in Figure 3. The distribution of diseases for the respective ranks per specialty is displayed in Table 1. The greatest range of diagnoses was encountered during the on call periods, where the three most common SMDs together accounted for only 25% of the total diagnoses. On the wards, the three most common SMDs ranged from 32% to 63% in the different subspecialties. As a general measure of diversity of SMDs we calculated how many of the most common diagnoses were needed to represent >75% of all diagnoses within a respective subspecialty. These were 15 (on call), 10 (nephrology, infectious diseases), six (cardiology), and five (endocrinology, gastroenterology, pulmonology, oncology/hematology). The percentage of subspecialty-specific main diagnoses per subspecialty is shown in Table 2. While on the cardiology wards 89% of the main diagnoses were diseases from the subspecialty of cardiology only 24% of diagnoses on the ward for infectious diseases were specific infectious diseases.

**Table 1:** **Most commonly encountered suspected main diagnoses**

Ward	1	2	3
**On call**	• Water and electrolyte imbalances	• Infections-special	---
		• Overdose/poisoning	
		• Acute renal failure	
**Nephrology**	• Urinary tract infection	• Respiratory tract infection	• Sepsis
			• Hypertension
			• Acute renal failure
**Endocrinology**	• Endocrine tumor	• Endocrine syndrome	• Tumor-unspecified
**Infectious diseases**	• Hepatitis/cirrhosis	• Infections-special	---
		• Gastroenteritis	
**Gastroenterology**	• Hepatitis/cirrhosis	• Gastrointestinal (GI) tumor	• Upper-GI-condition (unspecified)
**Oncology/hematology**	• Lymphoma	---	• Acute leukemia
	• Tumor-unspecified		• Chronic leukemia
**Pulmonology**	• Lung tumor	• COPD	• Pulmonary hypertension
**Cardiology**	• Stable coronary artery disease	• Valvular heart disease	• Acute coronary syndrome

**Table 2:** **Percentage of subspecialty-specific main diagnoses per subspecialty**

Subspecialty	Subspecialty-specific diagnoses
**Cardiology**	89%
**Pulmonology**	88%
**Gastroenterology**	86%
**Oncology/hematology**	77%
**Endocrinology**	68%
**Nephrology**	54%
**Infectious diseases**	24%

### Secondary diagnoses

Students were asked to document up to four relevant secondary diagnoses per patient on their PECs. We particularly looked for common general internal medicine diseases, in particular for: atrial fibrillation, anaemia, respiratory tract infections, COPD, diabetes, hypertension, hyperthyroidism, and renal insufficiency (Table 3). These diseases accounted for 7.7% (oncology/haematology) to 28.7% (pulmonology) of all documented relevant secondary diagnoses per specialty. Pulmonology, cardiology and nephrology showed the widest variety of these general internal medicine diseases while in endocrinology and oncology/hematology hardly any of these common general internal medicine diseases were documented as secondary diagnoses.

**Table 3:** **Common general internal medicine secondary diagnoses per subspecialty**

Secondary diagnosis	1	2	3	4	5	6	7
Atrial fibrillation (%)	17.16	---	4.26	---	5.95	3.85	6.02
Anaemia (%)	1.49	---	2.13	---	1.19	---	---
Respirat. tract infection (%)	0.75	---	---	5.26	---	---	4.82
COPD (%)	8.21	9.68	2.13	2.63	11.90	---	22.89
Diabetes (%)	11.94	---	17.02	10.53	13.10	---	12.05
Hypertension (%)	0.75	---	---	---	2.38	3.85	---
Hyperthyroidism (%)	1.49	---	---	---	3.75	---	3.61
Renal insufficiency (%)	9.70	---	4.26	2.63	16.67	---	6.02
**Total (%)**	**22.5**	**9.0**	**18.7**	**16.0**	**24.3**	**7.7**	**28.7**

1: cardiology, 2: endocrinology, 3: gastroenterology, 4: infectious diseases, 5: nephrology, 6: oncology/haematology, 7: pulmonology.

### Diagnostics and therapy

**Figure 4:**
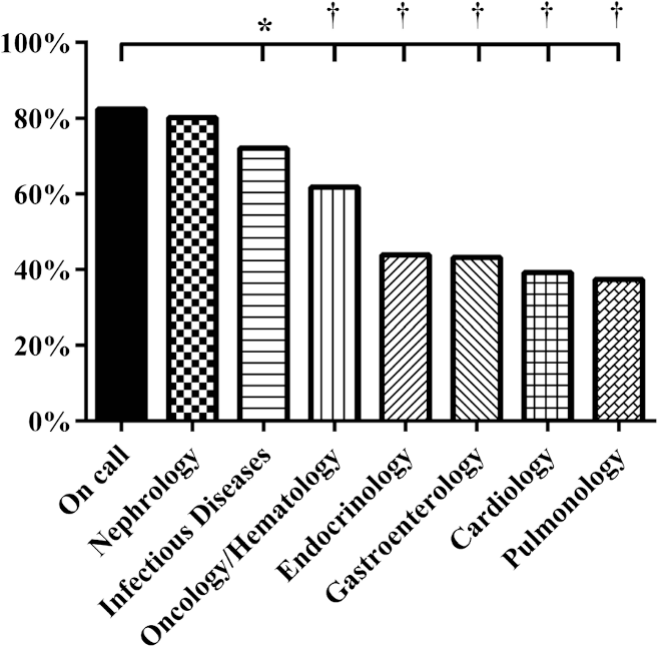
Relative frequency (%) of diagnostic procedures classified as basic, *p < 0.05, † p < 0.001.

**Figure 5:**
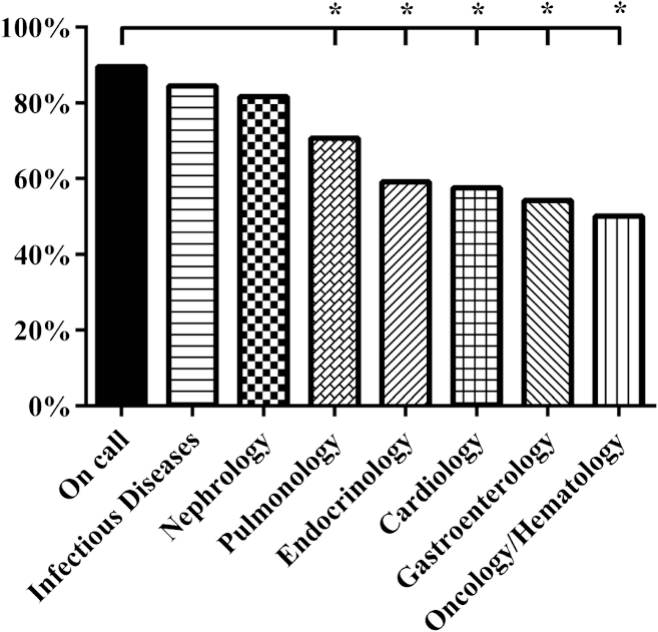
Relative frequency (%) of therapies classified as basic, *p < 0.001.

The percentage of all investigations that were classified as “basic” differed between the subspecialties, ranging from 82% (on call) to 37% (pulmonology) (Figure 4). No significant difference was found between on call and nephrology, the two subspecialties with the highest percentage of “basic” diagnostics. We found similar results for the percentage of “basic” therapy (Figure 5). The range for “basic” therapy was between 94% (on call) and 50% (oncology/hematology). Infectious diseases and nephrology were the two subspecialties, which showed no significant difference compared with on call.

## Discussion

Acquisition of general internal medicine knowledge and basic diagnostic, therapeutic and management skills are an important goal of internal medicine clerkships during undergraduate medical education. Students should be provided with relevant internal medicine assets, which will be useful independently of their further postgraduate training. Our findings suggest that the patient mix encountered by medical students during their final year internal medicine clerkship at a tertiary care center is narrow and specialized in most subspecialties. This reflects the increasing specialization and subspecialization in the field of internal medicine [9]. Interestingly, some subspecialties, in our study nephrology and also infectious diseases, provide a range of basic diagnostic and therapeutic skills which is as broad as during on call patient encounters. A survey of US internal medicine subspecialty fellows has shown that nephrology is being considered a very difficult subject matter to grasp [20]. This study and our data suggest that clinical thinking to choose the correct diagnostic and therapeutic measures seems to play a major role in this subspecialty resulting in a broader variety of procedures to be learned.

In other subspecialties, e.g. oncology/hematology, the three most common chief complaints and suspected main diagnoses accounted for 82% and 63% of the total, respectively. Furthermore, within a narrow spectrum of conditions, the students encountered a large percentage of rare disease. For example, in endocrinology, rare endocrine tumors and syndromes accounted for 31% and 18% of all diagnoses, respectively. This means that a student might spend the whole internal medicine clerkship without ever treating a patient with hypertension, pneumonia or any other common conditions of general internal medicine he will have to deal with even during postgraduate training in surgery. However, a study on the impact of subspecialty elective exposures on outcomes on the American board of internal medicine certification examination did not demonstrate significant positive associations between individual subspecialty elective exposures and ABIM-CE mean standardized passing score [16].

Despite these results for postgraduate training, we consider a greater patient variety to be relevant for the acquisition of general internal medicine knowledge during undergraduate internal medicine clerkships. Our data show, that a larger patient diversity with respect to diagnoses and to basic diagnostic and therapeutic procedures can be achieved by being exposed to learning experiences on call. Integrating on call rotations into internal medicine clerkships would be in line with the suggested core condition of “supported participation” for clinical workplace learning [21]. Hence, it should be considered to become a mandatory part of the curriculum for internal medicine clerkships. This would also be in line with the suggestions towards more competency-based medical education [22], which will have significant implications for the planning of internal medicine clerkships. Furthermore, other educational means, such as conference attendance and self-directed reading of an electronic knowledge resource [23] as well as multisource feedback [24] have been shown to be significantly associated with knowledge acquisition during internal medicine residency. These educational means might also support learning in undergraduate medical education.

In our setting, cardiology, pulmonology and gastroenterology showed the least variety in main diagnoses from the field of general internal medicine. However, clinical experiences with many different diseases during undergraduate training have been found to be important for expertise development in medical education with respect to knowledge encapsulation and illness script formation [25]. When we looked at a subset of common internal medicine conditions, we found that all students in all subspecialties encountered at least some of them as relevant secondary diagnoses. For students who encounter only a very limited number of specific main diagnoses in one subspecialty, teaching could focus also on secondary diagnoses of patients to broaden the spectrum of diseases encountered and to enhance clinical reasoning skills in students [26]. Furthermore, to expand the spectrum of problems to which students can be exposed, clinical teaching can be enriched by written clinical cases, simulated patients and other teaching methods, which should be made available to clinical teachers by the curricular planners [27].

In addition to including on call encounters in the internal medicine clerkship curriculum for final year undergraduate students, or to also focus teaching on secondary diagnoses from the field of general internal medicine, another option might be to shorten the rotations to four weeks per ward, which would give every student the opportunity to collect experiences from four different specialties. While subspecialty experience in internal medicine undergraduate training has been shown to be of value [28], rotating to four different wards will not solve the problem of encountering many patients with rare diseases or using very specialized tests or therapies rather than being exposed to more general internal medicine problems and basic diagnostic procedures and therapies while being on call. Additionally, with shorter rotations students will have to adjust to new nursing staff, new supervisors, and different working habits on new wards more frequently, which might impair their learning experience in the first couple of days. Our data show, that patients are admitted to the university hospital in several subspecialties with a diagnosis, which requires further specialized diagnostic or treatment. Many of these patients will have had more basic investigations or therapies in the community or smaller hospitals prior to admission. Thus, students at a university hospital will not have been engaged with this process. Much of the process of clinical decision-making and patient management, however, can be developed best by task-based learning [29] or by observing and forming an educational relationship with a role model [30]. Shadowing the resident on call would provide possibilities for both educational strategies and provide a broader understanding of general internal medicine problems. In addition, it exposes students to dealing with emergencies, which junior doctors find particularly challenging and feel ill prepared for by their undergraduate training [31].

One limitation of our study is the small number of participants. Although we collected 496 completed PECs from students in seven internal medicine subspecialties, some of these, for example endocrinology, only had a limited number of PECs (n = 31). Students were allocated their specific rotations according to choice, thus we cannot differentiate how much difference between the subspecialties is due to a difference in intrinsic motivation for a certain subspecialty and how much is due to differences between the students. Some students only completed very few PECs, which limits how well their PECs represented their learning environment. Finally, our study was limited to a single tertiary care center. Therefore, generalization of our findings might be difficult. However, our findings might still raise the awareness that certain problems regarding appropriate learning encounters for general internal medicine may exist.

## Conclusions

Internal medicine clerkships at a university hospital provide students with very limited patient diversity in many internal medicine subspecialties except nephrology. Shadowing the resident on call was associated with a much greater variety of common complaints, main suspected diagnoses, and basic diagnostic and therapy. To equip medical students with a broader diversity of general internal medicine skills at tertiary care centers, shorter rotations or on call learning opportunities should become a mandatory part of the final year internal medicine clerkship. Whether such curricular adaptations will also lead to better exam results needs to be investigated in further studies.

## References

[1] Muggah E, Graves E, Bennett C, Manuel DG (2012). The impact of multiple chronic diseases on ambulatory care use; a population based study in Ontario, Canada. BMC Health Serv Res.

[2] European Commission. 2009 Ageing Report. [http://ec.europa.eu/economy_finance/publications/publication14992_en.pdf]

[3] Song SO, Jung CH, Song YD, Park CY, Kwon HS, Cha BS (2014). Background and data configuration process of a nationwide population-based study using the korean national health insurance system. Diabetes Metab J.

[4] Pitkin AD, Rice MJ (2009). Challenges to glycemic measurement in the perioperative and critically ill patient: a review. J Diabetes Sci Technol.

[5] Ladjević N, Kalezić N, Ladjević IL, Vuksanović A, Durutović O, Jovanović D (2011). Preoperative assessment of patients with end stage renal failure. Acta Chir Iugosl.

[6] Domi R, Sula H, Ohri I, Begiri A, Kaci M, Bodeci A (2014). Anesthetic challenges of patients with cardiac comorbidities undergoing major urologic surgery. Int Arch Med.

[7] Mannucci PM, Nobili A, REPOSI Investigators (2014). Multimorbidity and polypharmacy in the elderly: lessons from REPOSI. Intern Emerg Med.

[8] James BC, Hammond ME (2000). The challenge of variation in medical practice. Arch Pathol Lab Med.

[9] Cassel CK, Reuben DB (2011). Specialization, subspecialization, and subsubspecialization in internal medicine. N Engl J Med.

[10] Howell JD (1989). The invention and development of American internal medicine. J Gen Intern Med.

[11] Abuhusain H, Chotirmall SH, Hamid N, O’Neill SJ (2009). Prepared for internship?. Ir Med J.

[12] Ochsmann EB, Zier U, Drexel H, Schmid K (2011). Well prepared for work? Junior doctors’ self-assessment after medical education. BMC Med Educ.

[13] Fletcher KE, Visotcky AM, Slagle JM, Tarima S, Weinger MB, Schapira MM (2012). The composition of intern work while on call. J Gen Intern Med.

[14] Approbationsordnung für Ärzte 07 01 2013. [http://www.gesetze-im-internet.de/_appro_2002/index.html#BJNR240500002 BJNE000200000]

[15] Rattner SL, Louis DZ, Rabowitz C, Gottlieb JE, Nasca TJ, Markham FW (2001). Documenting and comparing medical students’ clinical experiences. JAMA.

[16] Shanmugam VK, Tsagaris K, Schilling A, McNish S, Desale S, Mete M (2012). Impact of subspecialty elective exposures on outcomes on the American board of internal medicine certification examination. BMC Med Educ.

[17] Durning SJ, Artino A, Boulet J, La Rochelle J, Van der Vleuten C, Arze B (2012). The feasibility, reliability, and validity of a post-encounter form for evaluating clinical reasoning. Med Teach.

[18] UKE Lernzielkatalog. [http://www.uke.de/studierende/downloads/zg-studierende/pj_Lernzielkatalog.pdf]

[19] WHO ICD-10. [https://www.dimdi.de/static/de/klassi/icd-10-gm/kodesuche/onlinefassungen/htmlgm2014/index.htm]

[20] Jhaveri KD, Sparks MA, Shah HH, Khan S, Chawla A, Desai T (2013). Why not nephrology? A survey of US internal medicine subspecialty fellows. Am J Kidney Dis.

[21] Dornan T, Boshuizen H, King N, Scherpbier A (2007). Experience-based learning: a model linking the processes and outcomes of medical students’ workplace learning. Med Educ.

[22] Frank JR, Snell LS, Ten Cate O, Holmboe ES, Carraccio C, Swing SR (2010). Competency-based medical education: theory to practice. Med Teach.

[23] McDonald FS, Zeger SL, Kolars JC (2007). Factors associated with medical knowledge acquisition during internal medicine residency. J Gen Intern Med.

[24] Warm EJ, Schauer D, Revis B, Boex JR (2010). Multisource feedback in the ambulatory setting. J Grad Med Educ.

[25] Schmidt HG, Rikers RMJP (2007). How expertise develops in medicine: knowledge encapsulation and illness script formation. Med Educ.

[26] Eva KW (2004). What every teacher needs to know about clinical reasoning. Med Educ.

[27] Ramani S, Leinster S (2008). AMEE Guide no.34: teaching in the clinical environment. Med Teach.

[28] Al Kadri HM, Al-Moamary MS, Tamim HM, Al-Kadi MT (2012). Value of subspecialty experience in internal medicine undergraduate training. Saudi J Kidney Dis Transpl.

[29] Harden RM, Crosby J, Davis MH, Howie PW, Struthers AD (2000). Task-based learning: the answer to integration and problem-based learning in the clinical years. Med Educ.

[30] Lyon P (2004). A model of teaching and learning in the operating theatre. Med Educ.

[31] Illing JC, Morrow GM, Rothwell nee Kergon CR, Burford BC, Baldauf BK, Davies CL (2013). Perceptions of UK medical graduates’ preparedness for practice: a multi-centre qualitative study reflecting the importance of learning on the job. BMC Med Educ.

